# Protocol for Physiotherapy OR Tvt Randomised Efficacy Trial (PORTRET): a multicentre randomised controlled trial to assess the cost-effectiveness of the tension free vaginal tape versus pelvic floor muscle training in women with symptomatic moderate to severe stress urinary incontinence

**DOI:** 10.1186/1472-6874-9-24

**Published:** 2009-09-01

**Authors:** Julien Labrie, Yolanda van der Graaf, Eric Buskens, Stella ESM Tiersma, Huub CH van der Vaart

**Affiliations:** 1 Department of Obstetrics and Gynaecology, University Medical Centre Utrecht, The Netherlands; 2 Julius Centre for Health Sciences and Primary Care, University Medical Centre Utrecht, The Netherlands; 3 Department of Epidemiology, University Medical Centre Groningen, The Netherlands; 4 Department of Obstetrics and Gynaecology, VU University Medical Centre Amsterdam, The Netherlands

## Abstract

**Background:**

Stress urinary incontinence is a common condition affecting approximately 20% of adult women causing substantial individual (quality of life) and economic (119 million Euro/year spent on incontinence pads in the Netherlands) burden. Pelvic floor muscle training (PFMT) is regarded as first line treatment, but only 15-25% of women will be completely cured. Approximately 65% will report that their condition improved, but long term adherence to treatment is problematic. In addition, at longer term (2-15 years) follow-up 30-50% of patients will end up having surgery. From 1996 a minimal invasive surgical procedure, the Tension-free Vaginal Tape (TVT) has rapidly become the gold standard in surgical treatment of stress urinary incontinence. With TVT 65-95% of women are cured. However, approximately 3-6% of women will develop symptoms of an overactive bladder, resulting in reduced quality of life. Because of its efficacy the TVT appears to be preferable over PFMT but both treatments and their costs have not been compared head-to-head in a randomised clinical trial.

**Methods/Design:**

A multi-centre randomised controlled trial will be performed for women between 35 - 80 years old with moderate to severe, predominantly stress, urinary incontinence, who have not received specialised PFMT or previous anti-incontinence surgery. Women will be assigned to either PFMT by a specialised physiotherapist for a standard of 9-18 session in a period of 6 months, or TVT(O) surgery. The main endpoint of the study is the subjective improvement of urinary incontinence. As secondary outcome the objective cure will be assessed from history and clinical parameters. Subjective improvement in quality of life will be measured by generic (EQ-5D) and disease-specific (Urinary Distress Inventory and Incontinence Impact Questionnaire) quality of life instruments. The economical endpoint is short term (1 year) incremental cost-effectiveness in terms of costs per additional year free of urinary incontinence and costs per Quality Adjusted Life Years (QALY) gained. Finally, treatment strategy and patient characteristics will be combined in a prediction model, to allow for individual treatment decisions in future patients. Four hundred female patients will be recruited from over 30 hospitals in the Netherlands

**Trial registration:**

Nederlands trial register: NTR 1248

## Background

Urinary incontinence is a common problem among adult women, with an estimated overall prevalence of 40% and between 6-10% of women with severe incontinence.[1] Stress urinary incontinence is the predominant type of incontinence affecting approximately 50% of incontinent women. Thus, 20% of adult women will experience stress urinary incontinence. The severity of urinary incontinence is often expressed in the number of incontinence episodes/week, where 7-14 episodes/week is considered as moderate and more than 14 episodes/week as severe incontinence.[1]

In addition, urinary incontinence is known to have a negative impact on quality of life and the economical costs related to it are substantial. These patient-centred and economical outcomes are important. For the treatment of stress urinary incontinence there are two options: pelvic floor muscle training (PFMT) and surgery. PFMT is currently advised as primary treatment, because it is statistically significant more likely to improve incontinence symptoms as compared to no or sham physiotherapy (OR 1.42, 95%CI 1.28-1.58).[2] Approximately 65% of women treated with PFMT experience improvement of their incontinence. However, a 100% cure rate (no incontinence episodes) is recorded in only 15 to 28% of women.[3,4] In addition to the low percentage of objective cure, the long-term efficacy of PFMT is under debate. After a 3-15 year follow-up, 25-50% of women primarily treated with PFMT have undergone surgery.[5-7] This can be due to lack of persistent efficacy, but may also be related to lack of compliance with the physiotherapy program.

In the past decade, surgery for stress incontinence has made a huge progression with the introduction of minimally invasive surgical techniques. The Tension-free Vaginal Tape (TVT) and Tension-free Vaginal Tape Obturator (TVT-O) are the most frequently performed procedures. In both cases, a small incision is made under the mid urethra through which a polypropylene mesh is placed without tension under the urethra. If abdominal pressure increases, the urethra is compressed to the tape and continence is restored. An advantage of these procedures is that they can be performed in day-care.[8] After a TVT(O) procedure approximately 66% of women are completely cured (100% dry) and 80-95% of women show an improvement.[9] Furthermore, this effect seems to be longstanding. However, although minimal invasive, stress incontinence surgery carries a risk of complications. Major complications are extremely rare with an incidence of 0.007% for bowel perforation and 0.012% for vascular injury.[8] The most common complication of bladder perforation during the procedure (approximately 3-6% for TVT and below 1% for TVT-O is easily recognized during the procedure itself and carries no long-term morbidity. Adverse outcome of surgery in terms of the development of new symptoms may be a bigger concern. Voiding difficulties and symptoms of an overactive bladder are reported in 6% of cases.[9]

In the Netherlands, women are usually seen by the general practitioner (GP) first. The GP Dutch guidelines (revised version 2006) on stress incontinence advocate to either start treatment with PFMT with instructions of the GP, or to refer to a specialised physiotherapist. In 2001 the Health Counsel reported that only 1.6% of women were referred to a physiotherapist.[10] Thus, the majority of incontinent women were not referred to specialised therapy. Treatment by the GP consists of giving verbal and written instructions (as is described in their guideline) how to contract and train the pelvic floor, with first revision after 3 months. This is clearly different from the weekly, individual sessions given by a specialised (biofeedback) physiotherapist. If treatment by the GP fails, it is advocated to refer the woman for specialised PFMT physiotherapy. This is the group of women where our study aims at.

The main objective of our study is to answer the question whether it is more (cost) effective to refer for specialised PFMT or for TVT(O) surgery after the standard initial instruction and training given by the GP has failed.

Primary surgery in this group of patients may be cost-effective when compared to PFMT as its cure rate is higher. A comparison of both strategies has been performed in a small sample of patients comparing abdominal Burch surgical procedure versus PFMT.[11] Although this single study concluded that primary surgery was preferable, the design and power did not meet the standards to provide sufficient scientific evidence. Finally, it is unclear which factors are associated with success or failure of PFMT and TVT. After identification of these factors a prediction model can be built that can further individualize treatment. In addition to our cost-effectiveness analyses we aim to develop such a prediction model.

## Methods

### Study Aims

The primary aim of the PORTRET study is to compare the clinical and cost-effectiveness of PFMT versus the TVT/TVT-O procedure as treatment for moderate to severe stress urinary incontinence.

### Study design and setting

**Figure 1:**
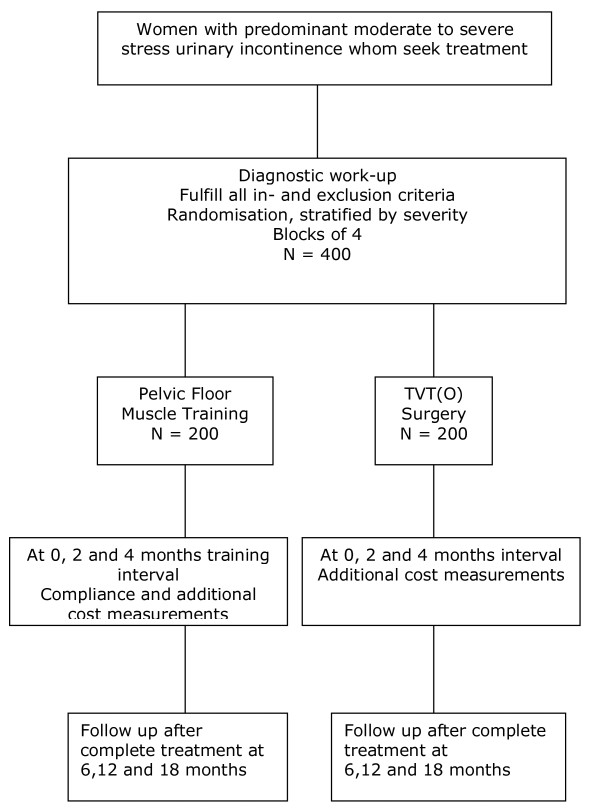
**Flow chart**.

The PORTRET study is a multidisciplinary, multicentre non-blinded therapeutic randomised controlled trial. Randomisation will be performed centrally with computerised randomisation tables. The two trial arms are PFMT by a specialised physiotherapist and a surgical intervention with the TVT or TVT-O. Recent data show that both the TVT and TVT-O are equally effective.[12,13] For obvious reasons blinding of treatment between PFMT and surgery will not be possible. To reflect daily practice as close as possible the assessment of costs will start directly after randomisation. We expect that the maximum effect of PFMT will be reached within 3-6 months. To account for possible changes in symptoms and changes in costs during this period, cost and symptom assessments are made every two months by means of a telephone administered questionnaire during the first 4 months. After that an assessment at 6, 12 and 18 months is performed. From literature, one of the predictive factors for the efficacy of treatment is the severity of incontinence at baseline. Therefore, we will introduce stratification by severity into the randomisation. We will stratify according to the severity (moderate or severe) as established by the Sandvik index [see table 1]. [14] The flowchart and study design are presented in figure 1 and table 2

**Table 1:** Sandvik Index

**SANDVIK INDEX**	Quantity of urine loss	
	
	1 = drops	2 = more than drops
Episodes		

1 = < 1 time a month	1	2

2 = 1 to more times a month	2	4

3 = 1 to more times a week	3	6

4 = daily	4	8

**Table 2:** Study Design Schedule

	Baseline visit	Randomisation	study entry	2, 4 months*	6 months visit	12 months visit	18 months visit
Patient characteristics	X	**Checking Inclusion & Exclusion Criteria**					
		
**Gynaecologic examination**	**X**					**X**	
		
Assessment of pelvic floor functioning	X				X	X	
		
**48 hour bladder diary**	**X**				**X**	**X**	
		
Flow	X				X	X	
		
**Residual volume**	**X**				**X**	**X**	
		
Stress test	X				X	X	
		
**Sandvik index**	**X**				**X**	**X**	
		
24 hour pad test	X					X	
		
**Global Impression of severity**	**X**			**X**	**X**	**X**	**X**
		
Global Impression of improvement				X	X	X	X
		
**EQ-5D**			**X**		**X**	**X**	**X**
		
UDI	X				X	X	X
		
**IIQ**	**X**				**X**	**X**	**X**
		
Costs assessment*			X	X	X	X	X
		
**Compliance with treatment #**				**X**		**X**	
		
Complications of surgery#				X			

* After randomisation costs assessment will be made for both groups at 2 and 4 months in order to account for changes

occurring during progression of (PFMT) treatment.

# Compliance is measured only for the PFMT group in order to assure proper training, complications are only related to surgery.

### Ethical consideration

The study has been approved for by the medical ethical review commission of the Universitary Medical Centre Utrecht (METC UMCU 07/278). Full medical ethical approval has been obtained on 05-02-2008.

### Identification and determination of eligible patients

All women aged 35-80 years whom present with symptomatic moderate to severe, according to the Sandvik severity index (≥ 3) [14], predominant stress urinary incontinence, will be considered for inclusion. They should present themselves with the problem of incontinence to their general practitioner, gynaecologist or urologist. They should not have already been referred for specialised pelvic floor muscle training.

The predominance of SUI is assessed with the Stress/Urge Incontinence Questionnaire (S/UIQ). Two questions are asked. "How many times in the last seven days have you had an accidental leakage of urine onto your clothing, underwear, or pad?".

- During an activity such as coughing, sneezing, laughing, running, exercising or lifting? Symptom of stress urinary incontinence (SUI).

- With a sudden strong need to pass water that you could not reach the toilet in time? Symptom of urge urinary incontinence (UUI).

For predominant stress urinary incontinence the number of SUI events should outnumber the number of UUI.

Urodynamic testing will not be used in this study, unless the physician decides this is necessary. Its sensitivity, specificity and predictive value for diagnosing and predicting outcome in female urinary incontinence has not been proved.[15] So no exclusion criteria will be based on urodynamic assessments (if performed).

SUI must have been demonstrated on physical examination or on urodynamic assessment if performed. Patients will be excluded in case of previous incontinence surgery, pelvic organ prolaps stadium 2 or higher according to POP-Q classification or in case of residual post voiding bladder volume of more than 100 ml on catheterisation or ultrasound (Bladderscan^®^)

### Study Interventions

After obtaining informed consent, eligible women will be assigned to either PFMT by a specialised physiotherapist for a standard of 9-18 sessions in a period of 6 months, or TVT(O) surgery.

### Randomisation

**Figure 2:**
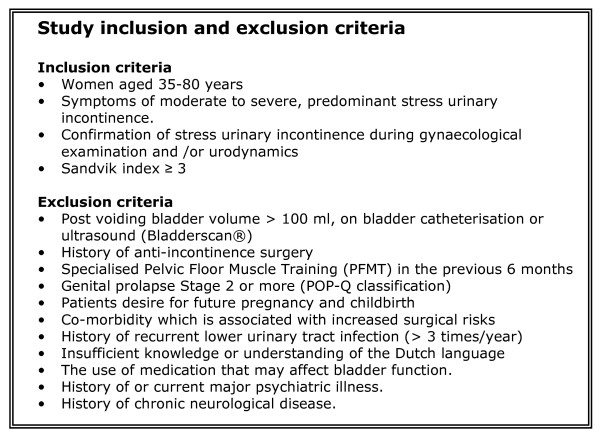
**Study inclusion and exclusion criteria**.

After checking the inclusion criteria an eligible patient is asked if she might be willing to participate and will receive written and verbal information about the study. A new appointment with the research nurse will be made. After an interval of one week the research nurse will ask if the patients wants to participate in the study and the exclusion criteria are checked. This will be done by the research nurse, not by the medical specialist him/herself. If an eligible patient wants to cooperate she is asked to sign the informed consent. When in- and exclusion criteria are met, randomisation will take place. Study inclusion and exclusion criteria are presented in figure 2.

Randomisation will be performed with the use of computerised randomisation tables. The randomisation will be stratified according to the severity of incontinence (moderate or severe as established by the Sandvik index. For obvious reasons (surgery versus conservative treatment) both the randomisation outcome and the personal involved in outcome assessment cannot be blinded.

### Patient follow-up procedures

Patients will be followed from the start of primary therapy until 18 months later. Follow up will consist of various elements

In all patients the next items will be recorded at inclusion:

1. Baseline characteristics

2. History and clinical examination

3. 48 h-bladder (voiding and incontinence) diary

4. 24 h-padtest

5. Validated Quality of Life questionnaires (Urinary Distress Inventory, Incontinence Impact Questionnaire, Euroqol 5D, patient global impression of severity/improvement)

6. Uroflow measurement including post void residual volume

7. Cost-analysis questionnaire

The onset of primary therapy, either TVT(O) in the surgery arm of the study or PFMT in the physiotherapy arm of the study will be T = 0

In case of surgery, the patient will receive the standard care associated with the pre-, per- and postoperative standards of the participating hospital. She will visit the hospital after surgery at 6 weeks, 6, 12 and 18 months. Questionnaires will be handed out at these visits containing the items mentioned above. Furthermore, special attention will be given with respect to extra visits to the general practioner, physiotherapist or specialist visits in relation to the surgical procedure that might have taken place and physical examination will be performed. Costs associated with incontinence and therapy will also be assessed with telephone administered questionnaire at 2 and 4 months follow-up. Women who are enrolled for PFMT will be followed-up according to the same schedule besides the 6 weeks post operative check-up.

### Patient outcome measures

This study will compare the clinical and cost-effectiveness of PFMT versus the TVT/TVT-O procedure as treatment for moderate to severe stress urinary incontinence.

Primary outcome: Subjective improvement of urinary incontinence

Secondary outcome(s):

1. Complete cure on objective and subjective parameters.

2. Subjective improvement in generic and disease-specific quality of life.

3. Complications and de novo urogenital symptoms.

4. The incremental cost-effectiveness of TVT as compared to PFMT, accounting for direct and indirect costs parameters.

5. Development of a prediction model for successful treatment, with the therapy given (PFMT or TVT) as independent variable in the model.

### Sample size considerations

Literature shows a subjective improvement of incontinence after PFMT up to 65%, and a subjective improvement after TVT(O) of at least 80%. In order to detect this difference with a power of 0.9 and an alpha of 0.05, we need 200 women in each trial arm. For our primary outcome parameter, total cure of incontinence, the expected difference between both arms is much larger: 28% cure after PFMT, and 65% cure after TVT. The 200 women in each treatment arm will be largely sufficient to assess a statistically significant difference in this secondary outcome.

Sample size calculation for the prediction models is based on the number of successes (or failures). For each predictor in the model we need 10-15 successes. Our pre-defined prediction model consists of 5 predictors and the interaction terms with treatment. Our total sample size will exist of 400 women. The expected number of successes (cure) of the PFMT group is 30 (15% of 200) women, the number of women cured after TVT is an estimated 130 (65% of 200 women). So the total number of complete cure is an estimated 160 women, with 240 women being not completely cured. These figures are sufficient for modelling up to 10-12 predefined possible predictors of cure. With respect to subjective improvement an estimated total of 290 women will show subjective improvement as measured with the Patient Global Impression of Improvement (PGI-I) questionnaire (65% of 200 women with PFMT plus 80% of 200 women in TVT group). Thus, 110 women can be considered failures. Again, this also allows us to make a prediction model for improvement with the predefined predictors. Therefore the study sample will also be amply sufficient for prediction purposes.

### Economic evaluation

In accordance with the primary aim of the clinical trial, the primary outcome for the economic evaluation will be the number of women with satisfactory results, i.e., subjectively improved, at 6, 12 and 18 months. During the period that women allocated to PFMT follow their training program, women whom underwent TVT(O) may already experience a period with satisfactory outcome. This time aspect will be accounted for by estimating and comparing person time with satisfactory results.

Health related quality of life (HRQoL) is considered a important outcome. In addition to the disease specific questionnaires (UDI and IIQ), two global questions, Patient Global Impression of Severity and Patient Global Impression of Improvement, (PGI-S and PGI-I), and a generic questionnaire, Euroqol 5D (EQ-5D) will be filled out at baseline and at regular intervals up to twelve months after completion of treatment. Using the EQ5D the outcome in terms of quality adjusted life years (QALYs) can be estimated. The effects observed by means of the disease specific questionnaire will be reported separately. [16-18]

Costs of the strategies directly TVT(O) as well as PFMT will be estimated using a societal perspective. Accordingly, direct medical costs will be based on the actual costs of the personnel and other resources used such as incontinence pads. Unit costs will be based on a specific costing study conducted in the hospital setting and the primary care setting. Where available unit costs will be based on the standard prices published in the current guidelines. Also, as PFMT is much more time consuming for the patients the costs associated to sick leave at work will be estimated using the friction cost method. The hours women spend on travel and training etc. will be assessed by questionnaires and subsequently valued.

#### COST-EFFECTIVENESS

The incremental costs per QALY gained will be estimated at one year. Uncertainty will be evaluated using standard bootstrap techniques (1,000 replicates). The incremental costs and effects will be depicted in a cost-effectiveness analysis (CEA) plane. The resulting 'scatter plot' provides information directly interpretable as the probability of one intervention being cost-effective compared to the alternative.

### Statistical analysis

Data will be presented as numbers (percentage) for nominal variables or means (standard deviation) for interval variables. Differences between the two interventions will be analysed based on intention-to-treat analysis. For interval variables, differences between the two groups will be analysed with a Students t-test, proportions will be compared using the Chi-square test.

Multivariate logistic regression analysis will be used for the development of the prediction of successful treatment only. In literature, a wide range of patient characteristics were considered to be associated with the outcome of PFMT. In the Consultation on Incontinence (ICI) proceedings it was concluded that there are no reliable factors identified yet, because the number of observations and studies is too low.[2] However, several factors have been identified as possible predictors of a successful outcome after PFMT. To provide information on treatment effectiveness in different subgroups of patients participating in this trial, we will derive a prediction model and estimate interaction of treatment effect with baseline incontinence severity (number of leaks/week), duration of symptoms, increasing parity, Body Mass Index, baseline pelvic floor contraction ability, and treatment modality as factors.[2] The defined prediction model will therefore be pre-specified resulting in less over-optimism than models in which the choice of predictors is strongly driven by the data-set and the need for external validation.[19] From the multivariate logistic model, in which success will be defined as cure (no objective or subjective incontinence) at 12 and 18 months follow-up, we will estimate the weight of the different coefficients. The modelling process will be internally validated by bootstrap resampling. This technique gives an impression of how "over-optimistic" the model is i.e., how much the performance of the model may deteriorate when applied to a new group of similar patients.[20]

### Time plan

Patient recruitment started in March 2008 and is planned to continue until December 2009. The follow-up period is 18 months, and therefore will continue until June 2011. The study is conducted in cooperation with the urogynaecology consortium of the Netherlands. Most participating clinics have disposition over a research nurse for follow-up and data collection in order to fill out the web-based case record forms.

### Knowledge transfer

Outcome of the PORTRET trial will be important for reconsidering the added value of prescribing PFMT to every patient with stress-incontinence as primary treatment, as well as comparing objective and subjective treatment success or failure between TVT and PFMT. The results of this study will be submitted to different international and national scientific associations such as the International Continence Society (ICS).

## Competing interests

The authors declare that they have no competing interests.

## Authors' contributions

HV was responsible for the identification of the lack in evidence considering sling surgery versus pelvic floor muscle training for urinary stress incontinence in women. YG and EB have contributed to the development of the protocol and study design. All authors read and approved the final manuscript

## Pre-publication history

The pre-publication history for this paper can be accessed here:


